# The role of child diets in the association between pre-pregnancy diets and childhood behavioural problems: a mediation analysis

**DOI:** 10.1017/S1368980022001410

**Published:** 2022-10

**Authors:** Dereje G Gete, Michael Waller, Gita D Mishra

**Affiliations:** Centre for Longitudinal and Life Course Research, School of Public Health, Faculty of Medicine, University of Queensland, 266 Herston Road, Brisbane, QLD 4006, Australia

**Keywords:** Pre-pregnancy diets, Childhood diets, Behavioural problems, Healthy Eating Index score, Principal component analysis, Mediation

## Abstract

**Objective:**

To quantify the mediating role of childhood diets in the relationship between maternal diets prior to pregnancy and childhood behavioural disorders.

**Design:**

The Healthy Eating Index score was constructed using a semi-quantitative and validated 101-item FFQ. We assessed childhood behavioural disorders using the Strengths and Difficulties Questionnaire. Three dietary patterns were identified using principal component analysis to explore childhood dietary patterns (high fats and sugar; prudent diets; and diary). A causal inference framework for mediation analysis was used to quantify the mediating role of childhood diets in the association between pre-pregnancy diets and the risk of offspring behavioural problems.

**Setting:**

This is a national representative population-based survey which covers all Australian citizens and permanent residents in Australia.

**Participants:**

We included 1448 mother–child pairs from the Australian Longitudinal Study on Women’s Health and its sub-study mothers and their children’s health.

**Results:**

We found a 20 % of the total effect of the poor adherence to pre-pregnancy diet quality on the risk of offspring behavioural problems was mediated through childhood high consumptions of fats and sugar. No clear mediating effect through prudent and diary childhood diets was observed.

**Conclusion:**

This study suggests that childhood high fats and sugar consumption may contribute to the total effects of the pre-pregnancy diets on the risk of childhood behavioural problems.

Childhood behavioural problems are the second leading cause of disease burden in young adolescents aged 10–14 years^(1)^. They have a substantial impact on adulthood productivity and function throughout the life course, including poor academic performance, occupational and psychosocial dysfunction^(2)^.

There is a growing body of evidence that recognises the importance of maternal diet quality on offspring behaviours^(3–5)^. Our previous study has shown that pre-pregnancy diet quality was also associated with lowering the risk of childhood behavioural problems^(6)^. Maternal diets may affect offspring behaviour through epigenetic changes and inflammation pathways^(7,8)^. Maternal diet quality has been positively linked with offspring diet quality^(9–11)^. In turn, childhood diet has been reported as a strong predictor for behavioural disorders^(12)^. Several studies showed that better adherence to childhood diet quality was associated with improving their behaviours or mental health^(13–15)^. To our knowledge, no studies, however, formally investigated the mediating role of childhood diets in the association between pre-pregnancy diets and offspring behaviours.

The Strengths and Difficulties Questionnaire (SDQ) has been widely used to assess childhood behavioural disorders. The SDQ total behavioural difficulties score comprises emotional, peer, conduct and hyperactivity subscales – which have been found to be a psychometrically sound measure of overall childhood behavioural problems in studies from around the globe^(16–18)^. A number of studies reported a high prevalence of childhood behavioural disorders assessed by SDQ^(19–21)^. In Australia, about 14 % of behavioural disorders were reported among children and adolescents aged 4–17 years^(22)^.

Studies over the past decade have provided key information on the association between maternal diets and offspring behavioural problems by adjusting maternal socio-demographic, lifestyle, perinatal and childhood factors^(23–25)^. An important unanswered issue, however, is whether the observed association is based on direct effects of maternal diet or indirect effects of childhood diet. A meta-analysis conducted by Borge et al.^(26)^ revealed that better quality of diets in pregnancy had a modest effect on improving offspring behaviours. The included studies in the meta-analysis controlled for childhood diet as a covariate in the adjusted model, though the observed associations were largely attenuated by childhood diets and suggesting that childhood diets might have a mediating effect in the association between maternal diets and offspring behaviours. The current study is, to our knowledge, one of the first large prospective cohort studies to use a casual inference frame to formally investigate the role of childhood diets in the association between pre-pregnancy diets and offspring behavioural problems. Identifying causal pathways in the association of maternal diets and childhood behavioural disorders could provide a better scientific basis for targeted prevention strategies.

The present study aimed to quantify the mediating role of child diets in the association between pre-pregnancy diet quality and childhood behavioural problems aged 5 to 12 years using a nationally representative cohort study of Australian mothers and their children.

## Methods

### Study participants and design

The current study used data from the Australian Longitudinal Study on Women’s Health (ALSWH) and Mothers’ and their Children’s Health (MatCH) study. For the ALSWH study, over 14 000 women born in 1973–1978 were recruited, who were randomly selected from the National Universal Health Insurance database, including all Australian citizens and permanent residents. Fuller details of the ALSWH have been described elsewhere^(27)^.

The MatCH is a substudy of the ALSWH young cohort, born 1973–1978, investigating childcare/school, illness/disability, diets, quality of life, social/emotional development, and growth and physical developments. In 2016, this study invited 8929 women to provide information about their children, among those, 3039 women filled out the questionnaire about their children (up to three young children) (*n* 5780)^(28)^.

**Fig. 1:**
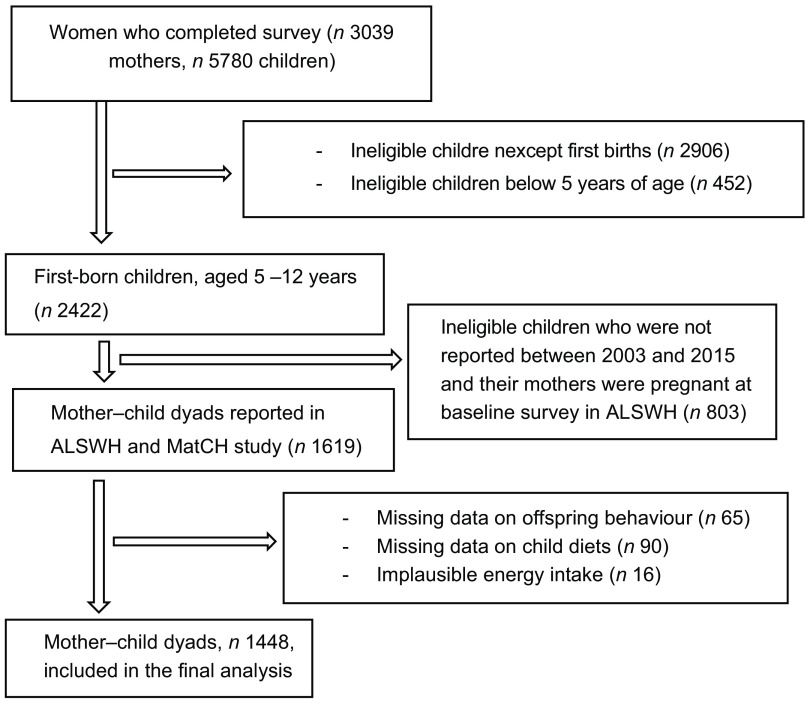
Flow chart of the final sample for the analysis of mediation by childhood diets in the association between pre-pregnancy diets and offspring behavioural problems

This study utilised data from the young Australian cohort, aged 18–23 years (born 1973–1978), who provided wide-ranging information about their offspring born between 2003 (Survey 3) and 2015 (Survey 7). Nulliparous and non-pregnant women at baseline Survey 3 and 5 were included in the study. Women who reported at least one live birth between Survey 3 and 7 were included. Only the first births born between 2003 and 2015 and were aged 5–12 years were included. We excluded women who had missing data on offspring behaviour, child diets and implausible energy intake (> 16 800 kJ/d or < 2100 kJ/d)^(29)^. The current study included 1448 mother–child dyads in the final analysis (Fig. 1). Data from the ALSWH were used to assess exposure (maternal dietary consumption), while the MatCH study was used to assess outcome (childhood behavioural problems) and mediator (childhood dietary consumption). The ALSWH study was approved by the Human Research Ethics Committees at the University of Newcastle and the University of Queensland, and informed consent was received from all ALSWH participants.

### Maternal dietary assessment

Women’s dietary consumption was first assessed in 2003 (Survey 3, aged 25–30 years, *n* 9081) and again in 2009 (Survey 5, aged 31–36, *n* 8200) for Epidemiologic Study version 2^(30)^. Women were asked to provide their habitual dietary intake of the previous 12 months using a validated and semi-quantitative 101-items FFQ. Validation of the FFQ against 7 d food diaries of sixty-three women of child-bearing age indicated moderate to strong energy-adjusted correlation coefficients for a wide range of nutrients and ranged from 0·28 for vitamin A to 0·78 for carbohydrates^(31)^.

We computed the Healthy Eating Index (HEI-2015) score to assess pre-pregnancy diet quality, which includes thirteen components that sum to a total maximum score of 100 points. Each of the dietary components is scored on a density basis out of 1000 calories except fatty acids^(32,33)^. Nine components, including total fruits, whole fruits, whole grains, total vegetables, greens and beans, dairy, seafood and plant proteins, total proteins, and fatty acids, were to be consumed adequately. However, four dietary components, such as refined grains, Na, saturated fats and added sugars, were to be consumed in moderation – in which mothers with lower intake receive higher scores. We categorised the HEI-2015 score was into the tertiles approach according to its distribution among the study participants: tertile 1 (low adherence), tertile 2 (moderate adherence) and tertile 3 (high adherence) to enable practical comparisons.

### Childhood dietary assessment

This study used a validated and semi-quantitative twenty-eight-items Children’s Dietary Questionnaire (CDQ) to assess childhood dietary patterns, which measures childhood dietary consumption either in the previous week or 24 h^(34)^. The CDQ was developed according to the most recent national data on the dietary consumption of Australian children^(35,36)^ and the Australian Dietary Guidelines^(37,38)^.

Children’s dietary patterns based on twenty-five non-overlapping food groups (frequency of consumptions/day) were identified using principal component analysis (PCA) with the use of orthogonal (varimax) rotation. The number of childhood dietary patterns was chosen with the basis of eigenvalues > 1·35, the identification of a breakpoint in the scree plot and factor interpretability^(39)^. We used the Bartlett test of sphericity (*P* < 0·001) to indicate statistically correlated variables. Kaiser–Meyer–Olkin test (0·71) was used to measure sampling adequacy. Food groups with factor loadings ≥ 0·30 on a factor were considered to have a strong association with that dietary pattern and be the most explanatory in describing the factors^(40)^.

### Offspring behaviour assessment

Women were asked to provide their offspring’s behaviours over the previous 6 months using the SDQ. Total behavioural difficulties score consists of four subscales, ranging from 0 to 40: emotional (somatic, unhappy, worried, nervous and fears), hyperactivity (distractible, restless, fidgety, reflective and attentive), peer (solitary, popular, good friend, bullied and prefers adult) and conduct problems (fights, tempers, obedient, steals and lies). Each subscale comprises five items with three-point response scales (0 for ‘not true’, 1 for ‘somewhat true’ and 2 for ‘certainly true’), with a subscale score ranging from 0 to 10^(16)^. Each one-point increase in the total behavioural difficulties score corresponds to an increase in the risk of developing a mental health disorder.

The data on the total behavioural difficulties score were skewed. We, therefore, dichotomised total behavioural problems score based on Goodman classifications^(16)^, comparing children with abnormal or borderline scores with children with normal scores. In this study, the total behavioural problem is defined according to the cut-off on the total behavioural difficulties score (≥ 14 on the maternal reports).

### Assessment of confounders and covariates

Baseline maternal confounders, such as marital status, education, income, smoking, alcohol intake and physical activity, were adjusted using the self-reported data before the index birth (Survey 3 or 5). Prenatal factors, including hypertensive disorder in pregnancy and gestational diabetes mellitus, were adjusted for using the same survey as the index birth (Survey 4–7).

Women’s alcohol consumption was classified as a non-drinker, low-risk drinker (≤ 14 drinks/week), risky drinker (15–28 drinks/week) and high-risk drinker (> 28 drinks/week)^(41)^. However, women with high-risk drinkers were merged with a risky drinker group due to very few women reported as high-risk drinkers (*n* 6, 0·42 %). We categorised the smoking status as never smoker, ex-smoker and current smoker^(42)^. Mothers were asked to provide only activity that lasted 10 min or more using a mailed physical activity questionnaire before recording daily pedometer step counts for seven consecutive days. A physical activity score was calculated according to frequency and duration of walking and moderate and vigorous-intensity activity and categorised as sedentary/low (< 600 total metabolic equivalents (MET) min/week), moderate (600–1200 MET min/week) or high (≥ 1200 MET min/week)^(43)^.

Mothers also were invited to provide information on their children’s sex, height, weight, history of premature birth (live birth < 37 weeks of gestation) and low birth weight (live birth weight < 2·5 kg), multiple births, and breast-feeding status in the MatCH study. Child BMI was calculated using their weight (kg)/height (m^2^), then categorised into underweight, normal, overweight and obese according to sex and age-specific BMI classifications for children^(44)^.

### Statistical analyses

Maternal and childhood characteristics were described according to adherence to pre-pregnancy HEI-2015 score and offspring behavioural problems. Mean differences were examined using *t* test, Pearson’s chi-square and ANOVA.

**Fig. 2:**
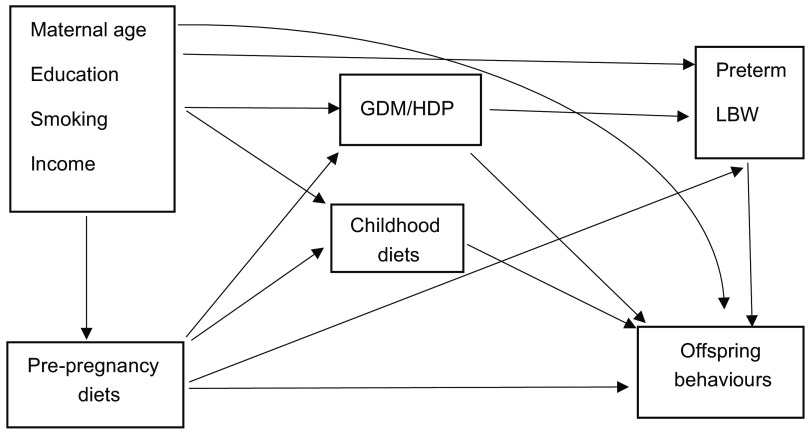
Directed acyclic graph showing potential pathways between pre-pregnancy diets, childhood diets and offspring behavioural problems

Figure 2 provides a directed acyclic graph indicating potential pathways between pre-pregnancy diets, childhood diets and offspring behavioural problems. A mediation analysis was performed using the counterfactual approach to decompose the total effect of poor pre-pregnancy diet quality on offspring behavioural problems into natural direct and indirect effects through childhood diets^(45,46)^. The mediation analysis was conducted by fitting a logistic regression model for the binary outcome (offspring behavioural problems, yes *v*. no) and a linear regression model for the continuous mediator (childhood diets). We did not include exposure–mediator interaction in any model, since it was not statistically significant (*P* > 0·05). From these combined models, we estimated OR of natural direct effects (OR^NDE^) and natural indirect effects (OR^NIE^), and total effects (OR^TE^) for the binary outcomes. The proportion mediated was calculated as (OR^NDE^ (OR^NIE^–1)] ÷ [OR^NDE^ × OR^NIE^–1) × 100 % for the binary outcome^(45,46)^. The proportion mediated estimates the extent to which the effect of the pre-pregnancy diets on the offspring’s behavioural problems is mediated through childhood diets relative to the overall effect of the pre-pregnancy diets. The maternal potential confounders were selected according to the known association from previous literature and then tested the associations with the exposure and outcome, the mediator and outcome, or/and the exposure and mediator. As shown in Table 2, two separate models were fitted to observe the difference of total proportion mediated by childhood diets after adjustments for baseline and post-covariates. The first model adjusted for baseline maternal potential confounders (maternal age, education, smoking and household income), since these variables might be associated with exposure, outcome and mediator. The second model further adjusted for pregnancy complications (gestational diabetes mellitus, hypertensive disorder in pregnancy and antenatal anxiety) and child characteristics (preterm birth, low birth weight, child age and sex). These variables are important post-covariates that might influence offspring behaviours (direct effect). We retained the confounders in the adjusted model if the *P*-value was < 0·2 in the simple model. We further performed a sensitivity analysis to observe changes in the HEI-2015 score from preconception to during pregnancy. We ran Spearman’s correlation and paired *t* tests to examine the stability and changes of the HEI-2015 score at the two time points. The paramed program in STATA version 16 (StataCorp) was employed to compute natural direct, indirect and total effects. *P*-value ≤ 0·05 was considered statistically significant.

## Results

The current study included 1448 mother–child dyads using the ALSWH and MatCH study (Fig. 1). A total of 198 (13·7 %) children experienced a higher SDQ score on total behavioural problems among the 1448 children between 2003 and 2015. The mean age of women at birth was 32 (sd 2·4) years.

The mean preconception HEI-2015 score was 58·6 (sd 12·3). Women had good adherence to total protein, fruits, added sugar, and greens and beans. However, they had low adherence to Na, fatty acids, seafood and plant proteins (online Supplementary Table 1).

Three children’s dietary patterns were identified from PCA from the MatCH survey with eigenvalues > 1·35 from scree plot and factor loadings, which explained 27 % of the total variation in food intakes. The first component was labelled ‘high fats and sugar’ and had high positive factor loadings for: potato chips/crisps or savoury biscuits; lollies, muesli or fruit bars; soft drink/cordial; ice cream/ice blocks; hot chips or French fries; and chocolates and takeaway. The second component ‘prudent diets’ had high positive factor loadings for bread and grains food, meat, fish, eggs, vegetables and fruits. The third component ‘diary’ had positive factor loadings for full cream/full-fat milk and regular yogurt/custard, and negative factor loading for reduced-fat milk, yogurt and custard (online Supplementary Table 2 and 3).

It can be seen from the data in Table 1 that a greater prevalence of offspring total behavioural problems was observed among women with lower educational status and income. There was also a higher proportion of offspring behavioural problems among obese women and antenatal anxiety. Women with better adherence to diet quality were older, were well-educated and did more physical activity. There was also a higher adherence to maternal diet quality among higher-income and urban residents.

**Table 1:** Maternal characteristics according to childhood behavioural problems aged 5–12 years and pre-pregnancy HEI-2015 score (*n* 1448)*

	Total behavioural problems				Pre-pregnancy HEI-2015 score									
Baseline maternal characteristics	Yes (*n* 198)			*P*-value†	Tertile 1 (*n* 483)			Tertile 2 (*n* 483)			Tertile 3 (*n* 482)			*P*-value†
	%	Mean	sd		%	Mean	sd	%	Mean	sd	%	Mean	sd	
Maternal age (years), mean (sd)		31·8	2·4	0·15		31·5	2·4		31·4	2·3		31·9	2·5	0·007
Marital status (%)‡				0·09										0·6
Married	12·9				32			35·4			32·5			
De facto/separated/divorced	11·9				35·5			31			33·5			
Single	16·8				32·9			32·9			34·2			
Residence (%)‡				0·26										0·01
Urban	13				31·1			33·6			35·3			
Rural/remote	15·2				38·1			32·6			29·2			
Education (%)‡				< 0·0001										0·001
Up to year 12 or equivalent	22·3				42·6			30·3			27			
Trade/apprenticeship/certificate/diploma	15·2				39·1			31·1			29·8			
University/higher degree	11·2				29·5			34·6			35·8			
Smoking (%)‡				0·18										0·004
Never smoked	12·8				32			34·8			33·2			
Ex-smoker	13·2				28·5			32·9			38·5			
Current smoker	17·3				42·7			28·1			29·2			
Alcohol intake (%)‡				0·28										0·07
Non-drinker	16·4				47·3			32·7			20			
Rarely drinker	16·7				35·5			35·5			28·9			
Low-risk drinker	13·3				32·3			32·6			35·1			
Risky drinker	7·7				28·8			42·3			28·8			
Physical activity (%)‡				0·58										< 0·0001
Sedentary/low, < 600 MET min/week	14·4				41·8			32·2			26			
Moderate, 600 to 1200 MET min/week	14·5				30·9			36·7			32·4			
High, ≥ 1200 MET min/week	12·5				27·5			32·2			40·2			
Pre-pregnancy BMI (%)‡				0·001										0·59
Healthy weight, < 25 kg/m^2^	12·2				34·3			32·7			32·9			
Overweight, 25–30 kg/m^2^	13·3				29·6			35			35·4			
Obese, ≥ 30 kg/m^2^	23·9				30·6			35·8			33·6			
Total calories intake (kJ/d), mean (sd)		1555	533·6	0·8		1740·9	688·7		1512·04	487·7		1385·0	453·7	< 0·0001
Household income (weekly) (%)‡				0·009										< 0·0001
≤ 999 $	17·4				47·1			28·9			24			
1000 $–1499 $	11·5				35·8			38			26·2			
≥ 1500 $	11·3				28·5			33·5			38			
Don’t know/don’t want to answer	20·2				29·8			30·7			39·5			
During pregnancy														
Gestational hypertension (%)‡				0·17										0·58
No	13·3				33			33·5			33·5			
Yes	17·3				37·2			31·4			31·4			
Gestational diabetes (%)‡				0·07										0·53
No	13·3				33·2			33·2			33·6			
Yes	20·5				38·5			33·3			28·2			
Antenatal anxiety (%)‡				0·008										0·65
No	13·3				33·2			33·4			33·3			
Yes	27·3				38·6			27·3			34·1			
Antenatal depression (%)‡				0·21										0·76
No	13·5				33·3			33·3			33·4			
Yes	21·2				39·4			30·3			30·3			

* Values are mean (sd) or percentage (%).

† *P*-values from Pearson’s chi-square, *t* tests or ANOVA.

‡ Missing values (marital status: *n* 2, residence: *n* 12, education: *n* 17, smoking: *n* 5, alcohol intake: *n* 4, physical activity: *n* 10, pre-pregnancy BMI: *n* 21, income: *n* 69, gestational hypertension: *n* 4, gestational diabetes: *n* 5, antenatal anxiety: 5 and antenatal depression: *n* 4). The HEI-2015 score was categorised as tertile 1 (low adherence), tertile 2 (moderate adherence) and tertile 3 (high adherence).

**Table 2:** Natural direct and indirect effects of the preconception diet quality on the risk of offspring behavioural problems and the proportion mediated through childhood dietary patterns

Offspring behavioral problems	OR ^NDE^ A direct effect of poor preconception HEI-2015 score (lowest *v*. highest tertile)*	95 %CI	OR ^NIE^ Mediated through child diets	95 %CI	OR ^TE^ Total effects of preconception HEI-2015 score	95 %CI	Proportion mediated by child diets (%)
Mediator: ‘High fats and sugar’ dietary patterns (per 1-sd increase)							
Model 1†	1·62	1·07, 2·44	1·10	1·03, 1·17	1·78	1·18, 2·68	21 %
Model 2‡	1·67	1·10, 2·54	1·10	1·03, 1·17	1·83	1·20, 2·78	20 %
Mediator: ‘Prudent’ dietary pattern (per 1-sd decrease)							
Model 1†	1·77	1·18, 2·66	1·01	0·97, 1·04	1·78	1·18, 2·67	2·2 %
Model 2‡	1·82	1·20, 2·76	1·01	0·97, 1·04	1·83	1·21, 2·77	2·1 %
Mediator: ‘Dairy’ dietary pattern (per 1-sd decrease)							
Model 1†	1·70	1·13, 2·56	1·05	0·98, 1·11	1·78	1·19, 2·68	11 %
Model 2‡	1·77	1·16, 2·69	1·04	0·97, 1·10	1·83	1·21, 2·78	8 %

NDE, natural direct effect; NIE, natural indirect effect; TE, total effect.

Model 1 was adjusted for baseline maternal confounders. Model 2 was adjusted for model 1 and further adjusted for pregnancy complications and childhood characteristics.

* OR for the effect of HEI-2015 score, lowest tertile compared with the highest tertile (reference group). A causal inference framework for mediation analysis was used to estimate OR and 95 % CI for total, natural direct and indirect effects. The natural direct and indirect effects were computed by fitting a logistic regression model for the binary outcome and a linear regression model for the continuous mediator. Total effects are equal to the product of the natural direct and indirect effects. The proportion mediated was calculated as (OR^NDE^ (OR^NIE^–1)) ÷ (OR^NDE^ × OR^NIE^–1) × 100 % and approximates the extent to which the effect of the exposure (preconception diets) on the outcome (childhood behavioural problems) is mediated through childhood diets relative to the overall effect of the exposure.

† Adjusted for maternal age, education, smoking and income.

‡ Adjusted for maternal age, education, smoking, income, gestational diabetes mellitus, hypertensive disorder in pregnancy, antenatal anxiety, preterm birth, low birth weight, child age, sex and number of siblings.

A significantly higher proportion of total behavioural problems was found among low birth weight, premature and overweight/obese children. Boys were more likely to experience behavioural problems. Behavioural problems were also more likely to present in children with greater consumption of fats and sugar-sweetened beverages, and lower consumption of fruit and vegetable (online Supplementary Table 4).

Table 2 presents the mediating role of childhood diets in the association between pre-pregnancy diet quality and childhood behavioural problems. Overall, poor maternal diet quality before pregnancy had significant natural direct and total causal effects on offspring behavioural problems. The natural direct effects were stronger than natural indirect effects. The total causal effect of the preconception diet quality that was mediated was 2 % and 8 % through childhood consumptions of prudent diets and dairy, respectively.

Interestingly, childhood dietary pattern characterised by high consumptions of fats and sugar had a significant natural indirect (mediated) effect in the relationship between poor maternal diet quality and childhood behavioural problems. The proportion of the total effect of lower adherence to the preconception diet quality on the risk of childhood behavioural problems (OR = 1·83, 95 % CI: 1·20, 2·78) that was mediated through childhood ‘high fats and sugar*’* consumptions (per 1-sd increase) was 20 % after adjustment for baseline maternal confounders, prenatal and childhood factors. For each model, a consistent mediated effect of childhood dietary pattern of ‘high fats and sugar’ was observed in the relationship between preconception diet quality and childhood behavioural problems at *P* < 0·006. However, no significant mediated effects were found for the childhood ‘prudent diets and dairy’ patterns.

From the data in Supplementary Table 5, the HEI-2015 score was quite stable from preconception to during pregnancy at *P* < 0·0001. Although a slight mean increment by 1·0 point in the HEI-2015 score was observed from preconception to during pregnancy, there were no statistically significant mean changes observed at the two time points (*P* = 0·06).

## Discussion

The current study set out with the aim of quantifying the mediating role of childhood diets in the association between maternal diet quality prior to pregnancy and childhood behavioural disorders. We found that a significant proportion of the total effect of pre-pregnancy diet quality on the risk of offspring behavioural disorders was mediated through childhood high consumptions of fats and sugar. Childhood diet comprising high consumptions of fats and sugar explained 20 % of the total effect of the poor adherence to preconception diet quality on the risk of offspring behavioural problems after adjustment for baseline maternal confounders, prenatal and childhood factors. No clear mediated effects were found through the childhood diet patterns labelled ‘prudent diet’ and ‘diary’.

To our knowledge, this is the first study to use a causal inference framework to examine the extent to which childhood diets contribute to the association between pre-pregnancy diets and the risk of childhood behavioural problems. Our previous study has shown the direct effects of preconception diet quality on the risk of childhood behavioural disorders^(6)^. However, the observed effect estimate was attenuated by childhood diets and suggesting that childhood diets might have a mediating role in the association between pre-pregnancy diets and offspring behaviours. No studies formally distinguished childhood diets as a distinct mediator or covariate in the association between preconception diets and offspring behaviours. Several reports showed an association between children’s dietary patterns and behavioural disorders, particularly attention-deficit hyperactivity disorder^(12,47,48)^. In the current study, children’s dietary pattern, especially high intake of fats and sugar, was also strongly associated with increased risk of behavioural problems. This may be explained by the fact that high fats and sugar consumptions have a substantial contribution to developing risk of obesity^(49)^, which have been linked with negative neuroplasticity changes, including hippocampal dysfunction^(50)^, oxidative stress and inflammation^(51)^, and subsequently affect mental health^(52)^. In contrast, Kohlboeck et al. demonstrated that grater adherence to childhood diet quality was associated with a lower risk of behavioural disorders^(13)^. In a prospective cohort study conducted on Australian adolescents, Jacka et al also observed a positive association between the higher score on healthful diets and better mental health^(14)^.

In the present study, there was a strong association between preconception diet quality and children’s dietary patterns. This finding was also reported in a Danish National Birth Cohort study, Ahrendt et al. showed a positive association between maternal diet quality and their offspring’s diet quality^(53)^. Accordingly, a potential association between maternal dietary patterns and children’s dietary intake could exist, eventually affecting later disease risk in offspring. Maternal dietary patterns before pregnancy are more likely to continue such habits during pregnancy and postnatally^(54)^, which will to some extent be reflected in childhood dietary habits, subsequently affecting offspring behaviours. Overall, childhood diet has been strongly linked with maternal diet and later risk of behavioural disorders. Childhood diet, therefore, may have an important role in the association between maternal diets and offspring behaviours.

Although childhood diets, particularly ‘high fats and sugar’ intake, had a substantial contribution to the total effect of the pre-pregnancy diets on the risk of childhood behavioural disorders as a single mediating variable, there might be other possible causal pathways that most of the effects are mediated through. The risk of childhood behavioural problems has been influenced by adverse pregnancy or birth outcomes, including gestational diabetes, hypertension and low birth weight. These variables might also contribute as mediators as well as intermediate confounders (mediator-outcome confounders affected by exposure) in the association between maternal diets and offspring behavioural problems. Further studies should be undertaken to examine other possible pathways by controlling intermediate confounders and their importance in explaining these associations.

The major strengths of this study are the nationally representative sample, population-based prospective cohort study, and comprehensive information on maternal and childhood factors. Another advantage is that our study formally examined a mediation analysis using a counterfactual approach allowing us to decompose the total effect into natural direct and indirect effects. A validated FFQ was used to assess women’s dietary intake, specifically designed for use in the Australian population. However, the current study was limited using self-report data on maternal dietary intake and their offspring’s behavioural problems, which might have recall and information bias. There might be residual and intermediate confounders that the study was unable to control for, though we adjusted for a wide range of maternal and childhood factors.

In conclusion, a childhood diet comprising high consumption of fat and sugar might have an important contribution to the total effect of the pre-pregnancy diets on the risk of childhood behavioural problems. This study, therefore, suggests that better maternal diet quality before pregnancy might improve offspring behaviours substantially through optimising the quality of diets in childhood. Our findings also highlight the important role of childhood diets in the association between maternal diets and enhancing the offspring behaviours. This study, therefore, supports that maternal and childhood diet quality may be important modifiable factors to improve childhood behaviours and quality of life.

## Supplementary material

For supplementary material accompanying this paper visit https://doi.org/10.1017/S1368980022001410. click here to view supplementary material

## References

[1] World Health Organization (2021) Adolescent Mental Health. https://www.who.int/news-room/fact-sheets/detail/adolescent-mental-health (accessed April 2021).

[2] Ogundele MO (2018) Behavioural and emotional disorders in childhood: a brief overview for paediatricians. World J Clin Pediatr 7, 9–26. 29456928 10.5409/wjcp.v7.i1.9 PMC5803568

[3] Steenweg-de Graaff J , Tiemeier H , Steegers-Theunissen RP et al. (2014) Maternal dietary patterns during pregnancy and child internalising and externalising problems. The Generation R Study. Clin Nutr 33, 115–121. 23541912 10.1016/j.clnu.2013.03.002

[4] Borge TC , Brantsæter AL , Caspersen IH et al. (2019) Estimating the strength of associations between prenatal diet quality and child developmental outcomes: results from a large prospective pregnancy cohort study. Am J Epidemiol 188, 1902–1912. 31375821 10.1093/aje/kwz166 PMC6825833

[5] Oddy WH , Robinson M , Ambrosini GL et al. (2009) The association between dietary patterns and mental health in early adolescence. Prev Med 49, 39–44. 19467256 10.1016/j.ypmed.2009.05.009

[6] Gete DG , Waller M & Mishra GD (2020) Pre-pregnancy diet quality and its association with offspring behavioral problems. Eur J Nutr 60, 503–515. 32405778 10.1007/s00394-020-02264-7

[7] Georgieff MK (2007) Nutrition and the developing brain: nutrient priorities and measurement. Am J Clin Nutr 85, 614S–620S. 17284765 10.1093/ajcn/85.2.614S

[8] Vucetic Z , Kimmel J , Totoki K et al. (2010) Maternal high-fat diet alters methylation and gene expression of dopamine and opioid-related genes. Endocrinology 151, 4756–4764. 20685869 10.1210/en.2010-0505 PMC2946145

[9] Ashman AM , Collins CE , Hure AJ et al. (2016) Maternal diet during early childhood, but not pregnancy, predicts diet quality and fruit and vegetable acceptance in offspring. Matern Child Nutr 12, 579–590. 25294406 10.1111/mcn.12151 PMC6860109

[10] Fisk CM , Crozier SR , Inskip HM et al. (2011) Influences on the quality of young children’s diets: the importance of maternal food choices. Br J Nutr 105, 287–296. 20807465 10.1017/S0007114510003302

[11] Emmett PM , Jones LR & Northstone K (2015) Dietary patterns in the Avon longitudinal study of parents and children. Nutr Rev 73, 207–230. 26395343 10.1093/nutrit/nuv055 PMC4586449

[12] Wiles NJ , Northstone K , Emmett P et al. (2009) ‘Junk food’diet and childhood behavioural problems: results from the ALSPAC cohort. Eur J Clin Nutr 63, 491–498. 18059416 10.1038/sj.ejcn.1602967 PMC2664919

[13] Kohlboeck G , Sausenthaler S , Standl M et al. (2012) Food intake, diet quality and behavioral problems in children: results from the GINI-plus/LISA-plus studies. Ann Nutr Metab 60, 247–256. 22677949 10.1159/000337552

[14] Jacka FN , Kremer PJ , Berk M et al. (2011) A prospective study of diet quality and mental health in adolescents. PLoS One 6, e24805. 21957462 10.1371/journal.pone.0024805 PMC3177848

[15] Dimov S , Mundy LK , Bayer JK et al. (2021) Diet quality and mental health problems in late childhood. Nutr Neurosci 24, 62–70. 30890044 10.1080/1028415X.2019.1592288

[16] Goodman R (1997) The strengths and difficulties questionnaire: a research note. J Child Psychol Psychiatr 38, 581–586. 10.1111/j.1469-7610.1997.tb01545.x 9255702

[17] Goodman A , Lamping DL & Ploubidis GB (2010) When to use broader internalising and externalising subscales instead of the hypothesised five subscales on the Strengths and Difficulties Questionnaire (SDQ): data from British parents, teachers and children. J Abnormal Child Psychol 38, 1179–1191. 10.1007/s10802-010-9434-x 20623175

[18] Cheng S , Keyes KM , Bitfoi A et al. (2018) Understanding parent–teacher agreement of the strengths and difficulties questionnaire (SDQ): comparison across seven European countries. Int J Meth Psychiatr Res 27, e1589. 10.1002/mpr.1589 PMC5937526 29024371

[19] Liu Q , Zhou Y , Xie X et al. (2021) The prevalence of behavioral problems among school-aged children in home quarantine during the COVID-19 pandemic in china. J Affect Disord 279, 412–416. 33099056 10.1016/j.jad.2020.10.008 PMC7543949

[20] D’Souza S , Underwood L , Peterson ER et al. (2019) Persistence and change in behavioural problems during early childhood. BMC Pediatr 19, 259. 31349812 10.1186/s12887-019-1631-3 PMC6659228

[21] D’Souza S , Waldie KE , Peterson ER et al. (2019) Antenatal and postnatal determinants of behavioural difficulties in early childhood: evidence from growing up in New Zealand. Child Psychiatr Hum Dev 50, 45–60. 10.1007/s10578-018-0816-6 29860616

[22] Australian Institute of Health and Welfare (2019) Mental Health Services in Australia. https://www.aihw.gov.au/reports/mental-health-services/mental-health-services-in-australia/report-contents/summary/prevalence-and-policies (accessed April 2019).

[23] Pina-Camacho L , Jensen S , Gaysina D et al. (2015) Maternal depression symptoms, unhealthy diet and child emotional–behavioural dysregulation. Psychol Med 45, 1851–1860. 25524365 10.1017/S0033291714002955

[24] Mesirow MS , Cecil C , Maughan B et al. (2017) Associations between prenatal and early childhood fish and processed food intake, conduct problems, and co-occurring difficulties. J Abnorm Child Psychol 45, 1039–1049. 27812905 10.1007/s10802-016-0224-y PMC5415431

[25] Gale CR , Robinson SM , Godfrey KM et al. (2008) Oily fish intake during pregnancy–association with lower hyperactivity but not with higher full-scale IQ in offspring. J Child Psychol Psychiatr 49, 1061–1068. 10.1111/j.1469-7610.2008.01908.x 18422546

[26] Borge TC , Aase H , Brantsæter AL et al. (2017) The importance of maternal diet quality during pregnancy on cognitive and behavioural outcomes in children: a systematic review and meta-analysis. BMJ Open 7, e016777. 10.1136/bmjopen-2017-016777 PMC5623570 28947450

[27] Dobson AJ , Hockey R , Brown WJ et al. (2015) Cohort profile update: Australian longitudinal study on women’s health. Int J Epidemiol 44, 1547.1547a–1547f. 26130741 10.1093/ije/dyv110

[28] Mishra GD , Moss K , Loos C et al. (2018) MatCH (Mothers and their Children’s Health) Profile: offspring of the 1973–1978 Cohort of the Australian Longitudinal Study on Women’s Health. Longitudinal Life Course Stud 9, 351–375.

[29] Willett W (1998) Nutritional Epidemiology. New York: Oxford University Press.

[30] Ireland P , Jolley D , Giles G et al. (1994) Development of the Melbourne FFQ: a food frequency questionnaire for use in an Australian prospective study involving an ethnically diverse cohort. Asia Pac J Clin Nutr 3, 19–31. 24351203

[31] Hodge A , Patterson AJ , Brown WJ et al. (2000) The Anti Cancer Council of Victoria FFQ: relative validity of nutrient intakes compared with weighed food records in young to middle-aged women in a study of iron supplementation. Aust New Zealand J Public Health 24, 576–583. 11215004 10.1111/j.1467-842x.2000.tb00520.x

[32] Krebs-Smith SM , Pannucci TE , Subar AF et al. (2018) Update of the Healthy Eating Index: HEI-2015. J Acad Nutr Diet 118, 1591–1602. 30146071 10.1016/j.jand.2018.05.021 PMC6719291

[33] Reedy J , Lerman JL , Krebs-Smith SM et al. (2018) Evaluation of the Healthy Eating Index-2015. J Acad Nutr Diet 118, 1622–1633. 30146073 10.1016/j.jand.2018.05.019 PMC6718954

[34] Magarey A , Golley R , Spurrier N et al. (2009) Reliability and validity of the Children’s Dietary Questionnaire; a new tool to measure children’s dietary patterns. Int J Pediatr Obes 4, 257–265. 19922040 10.3109/17477160902846161

[35] Bell A , Kremer P , Magarey A et al. (2005) Contribution of ‘noncore’foods and beverages to the energy intake and weight status of Australian children. Eur J Clin Nutr 59, 639–645. 15714218 10.1038/sj.ejcn.1602091

[36] Gehling R , Magarey A & Daniels LA (2005) Food-based recommendations to reduce fat intake: an evidence-based approach to the development of a family-focused child weight management programme. J Paediatr Child Health 41, 112–118. 15790321 10.1111/j.1440-1754.2005.00563.x

[37] Baghurst K , Binns C , Truswell A et al. (2003) Food for Health: Dietary Guidelines for Children and Adolescents in Australia. Canberra: Commonwealth of Australia.

[38] Smith A , Kellett E & Schmerlaib Y (1998) The Australian Guide to Healthy Eating. Canberra: Commonwealth Department of Health and Family Services.

[39] Kline P (2014) An Easy Guide to Factor Analysis. New York: Routledge.

[40] Yong AG & Pearce S (2013) A beginner’s guide to factor analysis: focusing on exploratory factor analysis. Tutorial Quant Meth Psychol 9, 79–94.

[41] National Health and Medical Research Council (2019) Australian Alcohol Guidelines: Health Risks and Benefits. https://books.google.com.au/books?id=D7-mAAAACAAJ (accessed June 2019).

[42] Australian Institute of Health and Welfare (2000) National Health Data Dictionary. Version 9. AIHW Catalogue no. HWI 24. Canberra: Australian Institute of Health and Welfare. https://www.aihw.gov.au/getmedia/aaeb3b0d-fe46-4284-bb89-e0dae4e9254b/nhdd09.pdf.aspx?inline=true (accessed June 2021).

[43] Brown WJ , Burton NW , Marshall AL et al. (2008) Reliability and validity of a modified self-administered version of the Active Australia physical activity survey in a sample of mid-age women. Aust N Z J Public Health 32, 535–541. 19076744 10.1111/j.1753-6405.2008.00305.x

[44] Cole TJ , Bellizzi MC , Flegal KM et al. (2000) Establishing a standard definition for child overweight and obesity worldwide: international survey. BMJ 320, 1240–1243. 10797032 10.1136/bmj.320.7244.1240 PMC27365

[45] VanderWeele T (2015) Mediation: Introduction and Regression-Based Approaches. Explanation in Causal Inference Methods for Mediation and Interaction. New York: Oxford University Press. pp. 20–97.

[46] VanderWeele TJ & Vansteelandt S (2010) Odds ratios for mediation analysis for a dichotomous outcome. Am J Epidemiol 172, 1339–1348. 21036955 10.1093/aje/kwq332 PMC2998205

[47] Woo HD , Kim DW , Hong Y-S et al. (2014) Dietary patterns in children with attention deficit/hyperactivity disorder (ADHD). Nutrients 6, 1539–1553. 24736898 10.3390/nu6041539 PMC4011050

[48] Park S , Cho SC , Hong YC et al. (2012) Association between dietary behaviors and attention-deficit/hyperactivity disorder and learning disabilities in school-aged children. Psychiatr Res 198, 468–476. 10.1016/j.psychres.2012.02.012 22999993

[49] Millar L , Rowland B , Nichols M et al. (2014) Relationship between raised BMI and sugar sweetened beverage and high fat food consumption among children. Obesity 22, E96–E103. 24318968 10.1002/oby.20665

[50] Kanoski SE & Davidson TL (2011) Western diet consumption and cognitive impairment: links to hippocampal dysfunction and obesity. Physiol Behav 103, 59–68. 21167850 10.1016/j.physbeh.2010.12.003 PMC3056912

[51] Makarem N , Bandera EV , Nicholson JM et al. (2018) Consumption of sugars, sugary foods, and sugary beverages in relation to cancer risk: a systematic review of longitudinal studies. Annu Rev Nutr 38, 17–39. 29801420 10.1146/annurev-nutr-082117-051805

[52] Peet M (2004) International variations in the outcome of schizophrenia and the prevalence of depression in relation to national dietary practices: an ecological analysis. Br J Psychiatr 184, 404–408. 10.1192/bjp.184.5.404 15123503

[53] Ahrendt Bjerregaard A , Halldorsson TI , Tetens I et al. (2019) Mother’s dietary quality during pregnancy and offspring’s dietary quality in adolescence: follow-up from a national birth cohort study of 19 582 mother–offspring pairs. PLoS Med 16, e1002911. 31513597 10.1371/journal.pmed.1002911 PMC6742222

[54] Cuco G , Fernandez-Ballart J , Sala J et al. (2006) Dietary patterns and associated lifestyles in preconception, pregnancy and postpartum. Eur J Clin Nutr 60, 364. 16340954 10.1038/sj.ejcn.1602324

