# Supplementary material for: The role of child diets in the association between pre-pregnancy diets and childhood behavioural problems: a mediation analysis
Source: Public Health Nutr. 2022 Jun 20;25(10):2876–85. doi: 10.1017/S1368980022001410 (PMC9991854; doi:10.1017/S1368980022001410)
Supplement: Supplementary file 1 [file S1368980022001410sup001.docx]

**The role of child diets in the association between pre-pregnancy diets and childhood behavioral problems: a mediational analysis**

Dereje G. Gete

**Supplementary Table 1.** Each component of pre-pregnancy HEI-2015 score with mean (SD) score and percentage (n= 1448)

| \| Components \| \| --- \| | Standard for  maximum score | Standard for minimum  score of zero | Maximum points | Mean (SD) | Percentage (%) |
| --- | --- | --- | --- | --- | --- | --- |
| Total HEI-2015 score |  |  | 100 | 58.6 (12.3) |  |
| **Adequacy:** |  |  |  |  |  |
| Total Fruits | > 0.8 C eq./1,000 kcal | No fruit | 5 | 4.3 (1.2) | 86 |
| Whole Fruits | >0.4 C  eq./1,000 kcal | No whole fruit | 5 | 4.3 (1.1) | 86 |
| Total Vegetables | >1.1 C eq./1,000 kcal | No vegetables | 5 | 3.6 (1.3) | 72 |
| Greens and Beans | >0.2 C eq./1,000 kcal | No Greens and Beans | 5 | 4.7 (0.7) | 94 |
| Whole Grains | >1.5 oz. eq./1,000 kcal | No whole grains | 10 | 5.1 (4.1) | 51 |
| Dairy | >1.3 C eq./1,000 kcal | No dairy | 10 | 5.6 (3.3) | 56 |
| Total Protein | >2.5 oz. eq./1,000 kcal | No protein foods | 5 | 4.8 (0.6) | 96 |
| Seafood and Plant Proteins | >0.8 C eq./1,000 kcal | No seafood or plant proteins | 5 | 1.6 (1.04) | 32 |
| Fatty Acids | (PUFA + MUFA)/SFA (g/day) > 2.5 | (PUFA + MUFA)/SFA (g/day) <1.2 | 10 | 2.8 (3.1) | 28 |
| **Moderation:** |  |  |  |  |  |
| Refined Grains | <1.8 oz. eq./1,000 kcal | >4.3 oz. eq./1,000 kcal | 10 | 6.4 (3.4) | 64 |
| Sodium | <1.1 g/1,000 kcal | >2.0 g/1,000 kcal | 10 | 0.04 (0.6) | 0.4 |
| Added Sugars | <6.5% of energy | >26% of energy | 10 | 9.6 (1.4) | 96 |
| Saturated Fats | <8% of energy | >16% of energy | 10 | 5.5 (3.9) | 55 |

Polyunsaturated fatty acids (PUFAs), monounsaturated fatty acids (MUFAs), saturated fatty acids (SFAs), cup (C), ounce (oz.).

**Supplementary Table 2.** Factor loading matrix for the three childhood dietary patterns extracted with the use of 25 non-overlapping food groups (n= 1448) ^a^

| **Child dietary patterns** | **Factor loading** | | **Variance Explained (%)** |
| --- | --- | --- | --- |
| **High fats and sugar** |  | | 12 % |
| Potato chips/crisps or savory biscuits | 0.37 | |  |
| Take-away ^b^ | 0.33 | |  |
| Soft drink/cordial | 0.32 | |  |
| French fries | 0.31 | |  |
| Ice-cream/ice blocks | 0.31 | |  |
| lollies, muesli, or fruit bars | 0.31 | |  |
| Chocolates | 0.30 | |  |
| **Prudent diets** | |  | 21% |
| Vegetables (raw or cooked) | | 0.42 |  |
| Fruits | | 0.41 |  |
| Bread and grain (cereals) | | 0.38 |  |
| Cheese and/or cheese spreads | | 0.37 |  |
| Meat, fish, and eggs | | 0.32 |  |
| **Dairy** | |  | 27% |
| Full cream/full-fat milk | | 0.56 |  |
| Regular yogurt | | 0.33 |  |
| Reduced-fat milk | | -0.57 |  |
| Reduced fat/ low fat yogurt/ custard | | -0.34 |  |

^a^ Values are correlation coefficients between each food group and the dietary pattern derived from PCA. Absolute values < 0.30 and – 0.30 were not listed. High fats and sugar; prudent diets; and dairy. ^b^ Take-away: McDonald's, KFC, fish, and chips/ chicken shop)

**Supplementary Table 3.** Factor loading coefficients for all 25 non-overlapping food groups (n= 1448) ^a^

| **Childhood dietary patterns** | **Component 1**  **High fats and sugar** | **Component 2**  **Prudent diets** | **Component 3**  **Dairy** |
| --- | --- | --- | --- |
| Bread and grain (cereals) | 0.06 | **0.38** | -0.08 |
| Meat, fish, and eggs | 0.04 | **0.32** | 0.04 |
| Non-meat (tofu, nuts, and seeds) | -0.09 | 0.24 | 0.08 |
| Fruit juice | 0.20 | -0.03 | 0.16 |
| Water | -0.06 | 0.26 | -0.09 |
| Full cream/full-fat milk | 0.02 | 0.14 | **0.56** |
| Reduced-fat milk | 0.02 | 0.12 | **-0.57** |
| Cheese and/or cheese spreads | 0.05 | **0.37** | 0.07 |
| Regular yogurt | -0.01 | 0.18 | **0.33** |
| Reduced fat/ low fat yogurt/ custard | 0.04 | 0.10 | **-0.34** |
| Vegetables (raw or cooked) | -0.07 | **0.42** | 0.04 |
| Fruits | -0.01 | **0.42** | 0.01 |
| Peanut butter | 0.04 | 0.08 | 0.02 |
| Pre-sugared cereals (coco pops, fruit loops) | 0.19 | -0.02 | 0.12 |
| Sweet biscuits, cakes, muffin, and doughnuts | 0.28 | 0.11 | -0.10 |
| Potato chips/crisps or savory biscuits | **0.37** | 0.04 | -0.08 |
| lollies, muesli, or fruit bars | **0.31** | 0.08 | -0.04 |
| Chocolates | **0.30** | 0.08 | -0.08 |
| Soft drink/cordial | **0.32** | -0.10 | 0.08 |
| Ice-cream/ice blocks | **0.31** | 0.00 | -0.02 |
| Pie, pasty or sausage roll | 0.18 | -0.04 | 0.08 |
| Pizza | 0.15 | -0.01 | 0.04 |
| French fries | **0.31** | -0.08 | 0.06 |
| Hot dog, sausage, processed meat | 0.19 | 0.07 | -0.15 |
| Takeaway ^b^ | **0.33** | -0.08 | 0.07 |
| Variance Explained (%) | 12% | 21% | 27% |

^a^ Values are correlation coefficients between each food group and the dietary pattern derived from PCA. Factor loading coeficients above 0.30 are shown in bold. ^b^ Take-away: McDonald's, KFC, fish, and chips/ chicken shop)

**Supplementary Table 4.** Childhood characteristics according to total behavioral problems (n=1448) ^a^

| **Child characteristics** | **Child total behavioral problems** | | ***p-*value ^b^** |
| --- | --- | --- | --- |
|  | **No (n= 1250)** | **Yes (n= 198)** |  |
| Preterm birth (%) ^c^  No  Yes | 87.1  78.2 | 12.9  21.8 | 0.006 |
| Low birth weight (%) ^c^  No  Yes | 86.9  77.9 | 13.1  22.1 | 0.01 |
| Child age (years), mean (SD) | 8.7 (0.1) | 8.3 (0.1) | 0.004 |
| Child sex (%)  Male  Female | 83.7  89.4 | 16.3  10.6 | 0.002 |
| Child body mass index (%)  Underweight  Normal  Overweight  Obese | 82.5  87.7  79.0  83.3 | 17.5  12.2  21.0  16.7 | 0.02 |
| Breastfeeding status (%) ^c^  Never received  Received | 91.9  86.2 | 8.1  13.7 | 0.32 |
| Multiple births (%) ^c^  No  Yes | 86.5  81.1 | 13.5  18.9 | 0.35 |
| Number of siblings (%) ^c^  None  One  Two | 79.1  87.1  87.6 | 20.9  12.9  12.4 | 0.02 |

^a^ Values are mean (SD) or %, ^b^ *p*- values from Pearson chi-square or t- tests, ^c^ missing values (preterm birth: n= 6, breast feeding status: n= 29, multiple birth: n= 66).

**Supplementary Table 5.** Changes in HEI-2015 scores from preconception to during pregnancy (n= 621) ^a^

| Dietary patterns | Time points  (n= 621) | Mean (SD) | Spearman’s correlation coefficient (r) |
| --- | --- | --- | --- |
| HEI-2015 score | Preconception  During pregnancy  Mean difference | 58.4 (12.5)  59.4 (10.9)  1.0 (12.7) | 0.4 |
|  | *p-*value ^b^ | 0.06 | < 0.0001 |

^a^ Values are mean (SD) and correlation coefficients (r), ^b^ *p*-values from and paired t-test and Spearman’s correlation test.
